# DeeP4med: deep learning for P4 medicine to predict normal and cancer transcriptome in multiple human tissues

**DOI:** 10.1186/s12859-023-05400-2

**Published:** 2023-07-04

**Authors:** Roohallah Mahdi-Esferizi, Behnaz Haji Molla Hoseyni, Amir Mehrpanah, Yazdan Golzade, Ali Najafi, Fatemeh Elahian, Amin Zadeh Shirazi, Guillermo A. Gomez, Shahram Tahmasebian

**Affiliations:** 1 Present Address: Department of Medical Biotechnology, School of Advanced Technologies, Shahrekord University of Medical Sciences, Shahrekord, Iran; 2 Laboratory of Systems Biology and Bioinformatics (LBB), University of Tehran, Tehran, Iran; 3 Faculty of Mathematics, Shahid Beheshti University, Tehran, Iran; 4 Department of Mathematics, Faculty of Basic Sciences, Iran University of Science and Technology,(IUST), Tehran, Iran; 5 Molecular Biology Research Center, Systems Biology and Poisonings Institute, Baqiyatallah University of Medical Sciences, Tehran, Iran; 6 Centre for Cancer Biology, SA Pathology and University of South Australia, Adelaide, SA 5000 Australia; 7 Cellular and Molecular Research Center, Basic Health Sciences Institute, Shahrekord University of Medical Sciences, Shahrekord, Iran

**Keywords:** P4 medicine, Deep learning, Gene expression matrix, Prediction model, Classification, Tumor

## Abstract

**Background:**

P4 medicine (predict, prevent, personalize, and participate) is a new approach to diagnosing and predicting diseases on a patient-by-patient basis. For the prevention and treatment of diseases, prediction plays a fundamental role. One of the intelligent strategies is the design of deep learning models that can predict the state of the disease using gene expression data.

**Results:**

We create an autoencoder deep learning model called DeeP4med, including a Classifier and a Transferor that predicts cancer's gene expression (mRNA) matrix from its matched normal sample and vice versa. The range of the F1 score of the model, depending on tissue type in the Classifier, is from 0.935 to 0.999 and in Transferor from 0.944 to 0.999. The accuracy of DeeP4med for tissue and disease classification was 0.986 and 0.992, respectively, which performed better compared to seven classic machine learning models (Support Vector Classifier, Logistic Regression, Linear Discriminant Analysis, Naive Bayes, Decision Tree, Random Forest, K Nearest Neighbors).

**Conclusions:**

Based on the idea of DeeP4med, by having the gene expression matrix of a normal tissue, we can predict its tumor gene expression matrix and, in this way, find effective genes in transforming a normal tissue into a tumor tissue. Results of Differentially Expressed Genes (DEGs) and enrichment analysis on the predicted matrices for 13 types of cancer showed a good correlation with the literature and biological databases. This led that by using the gene expression matrix, to train the model with features of each person in a normal and cancer state, this model could predict diagnosis based on gene expression data from healthy tissue and be used to identify possible therapeutic interventions for those patients.

**Supplementary Information:**

The online version contains supplementary material available at 10.1186/s12859-023-05400-2.

## Background

In the past, diseases were considered the result of alterations in the function of one or more genes, so the diagnosis and treatment of patients were based on reductionist approaches to correct these genetic alterations. However, a fundamental revolution in medicine is a change from this reductionist view to a more holistic (systems biology) approach to understanding the biology of disease [1–3]. In systems biology, organ function results from the simultaneous interaction of all genes, mRNA, proteins, and metabolites across different cells constituting different types of tissues. Therefore, omics studies aimed to collect High-throughput genomic, epigenomic, transcriptomic, proteomic, and metabolomic data [4]. From this perspective, each omics dataset is a network layer, and the cell was considered as several integrated networks, so the disease is defined as a disorder or change in these networks [5].

P4 medicine (predict, prevent, personalize, and participate) is the latest approach to overcoming complex diseases like cancer. The development of computational models that can use omics data to predict disease and offer proper drugs to each person is very challenging [6, 7]. One of the essential omics is transcriptomic and deep learning is a powerful method for processing gene expression data and extracting new knowledge from disease [8]. In 2019, Lotfollahi developed scGen to analyze and predict the effect of a perturbation (i.e., drug, disease) at single-cell resolution [9]. This was followed by several review articles that explained the role of data science and machine learning in precision medicine (Fröhlich in 2018, Papadakis in 2019, and MacEachern in 2020 published) [10–12]. Finally, in 2022, Leon Hetzel developed a deep learning model for drug discovery based on cellular response to perturbations in a single-cell transcriptomics context [13]. Also, many research consortia worldwide have started working in this field, including MLPM (Machine learning for personalized medicine) at the Marie Curie Initial Training Network, funded by the European Union [14–17].

Many studies aimed to obtain genes expressed differently in tumors and normal. These genes are critical to understanding the function of the disease, but in these studies, two groups of individuals were compared [18–20]. At the same time, cancer is a complex disease, and patients with the same type of cancer may have different gene expressions. Also, some studies were performed to repurpose drugs for diseases based on these genes [21], but one drug is effective in some patients and ineffective in others. Our goal is to get one step closer to personalized medicine by trying to get the cancer-related genes for each person individually. So We try to make every tumor sample as close to normal as possible to find effective genes specific to that patient.

**Fig. 1:**
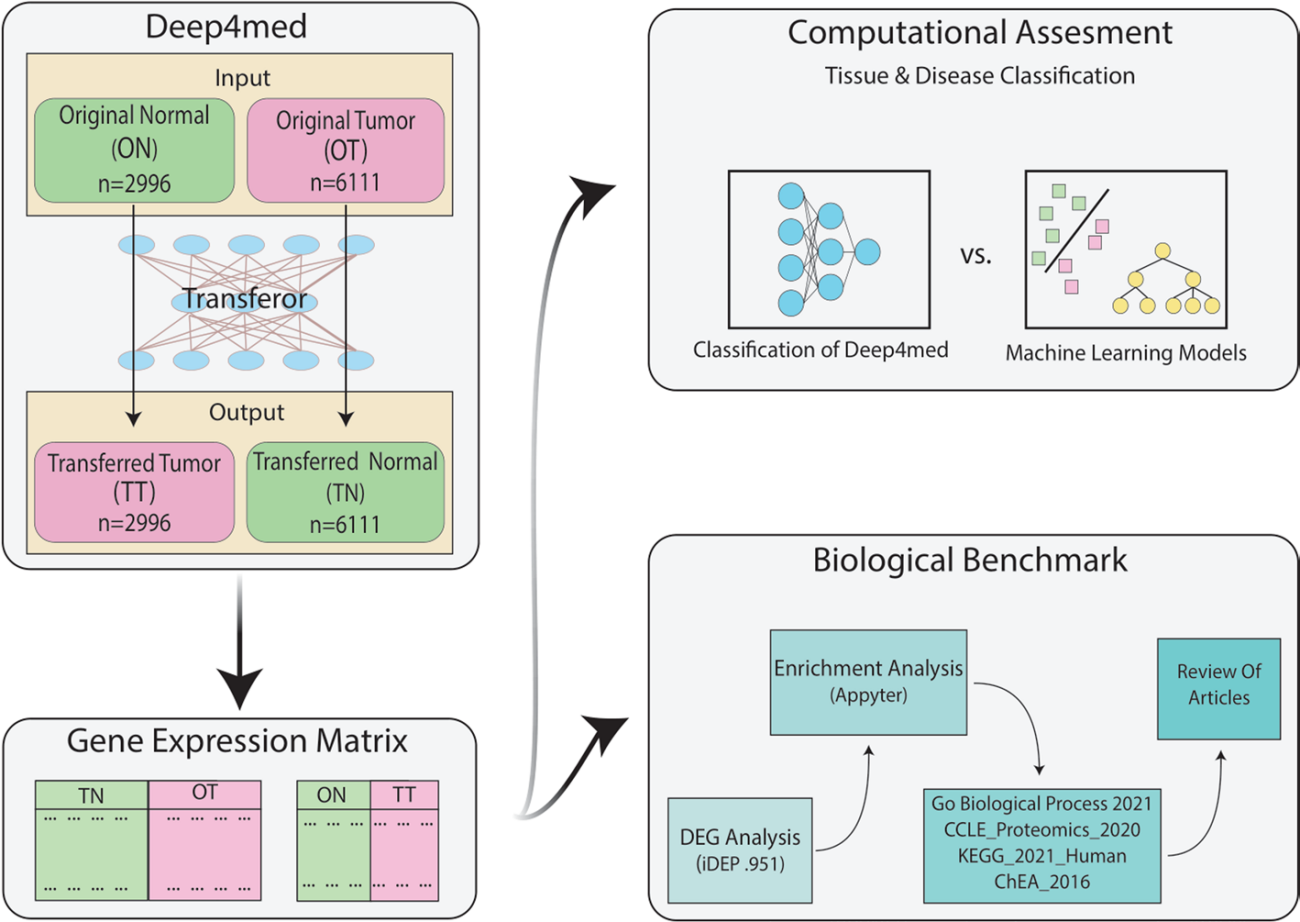
Workflow of our article: In the first step, we convert the tumor samples to the closest possible normal ones and the normal ones to the closest possible tumor samples and obtain new gene expression matrices. To evaluate the performance of our model through computational models, we compare the classification accuracy of our model with other machine learning models. Finally, we evaluate the obtained biomarkers through benchmark biology

In this study, we developed a model called DeeP4med to apply deep learning in P4 medicine. We used the datasets collected and preprocessed [22] which is a combination of The Cancer Genome Atlas (TCGA) [23] and genotype-tissue expression project (GTEx) [24]. This dataset contains 6111 tumor and 2996 normal samples in total that have been sampled from 13 different tissues. We selected 18,154 features (genes) that were common across all samples. For simplicity, we ignored the sub-tissue classification within the tissue type. In the preprocessing step, we divided each feature by its maximum value in the dataset. DeeP4med comprises a classifier and a Transferor. The classifier is used to identify the tissue type and the tissue condition (normal or tumor). Transferor takes a person's normal expression matrix and predicts the tumor matrix in the same person and vice versa. Hence, the model tries to learn the important features of converting a normal sample to a tumor sample. Then, based on a sample's important features and other personal features, it predicts and generates a new expression matrix. Because of this ability of the model, it considers two components of P4 medicine: predict and personalize, and by using them, we can achieve two other components: prevent and participate. To evaluate the results predicted by the model, we analyzed them in terms of conventional machine learning and bioinformatics methods, which are reviewed in the results section. (Fig. 1.)

## Results

After creating the model, we evaluated the model’s performance with two different approaches: (1) Performance analysis of the Transferor and Classifier of DeeP4med. (2) Investigating the validity and biological significance of the data generated by the model by Differentially expressed genes (DEGs) and enrichment of analysis.

### Performance analysis of transferor and classifier

**Fig. 2:**
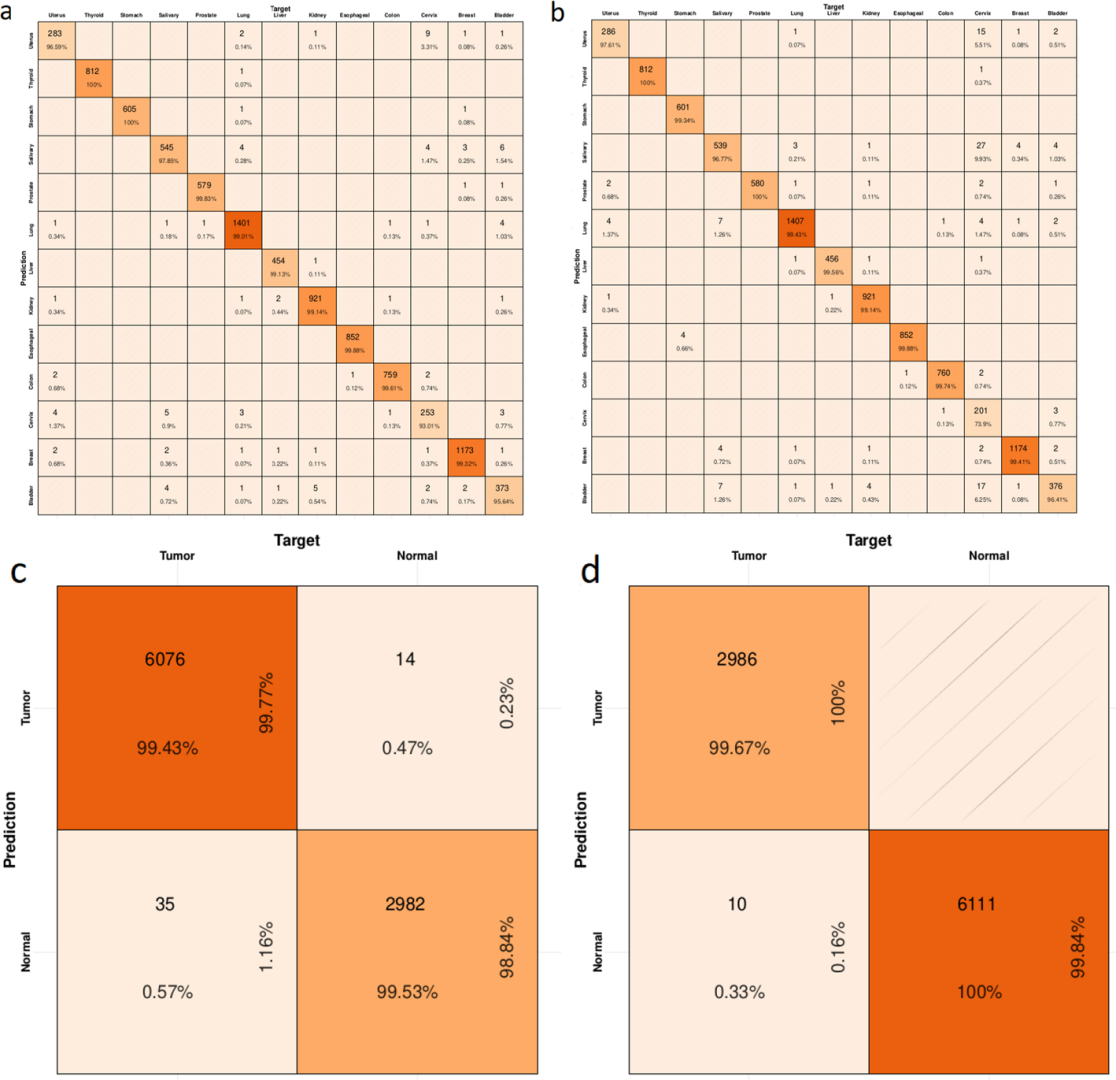
The confusion matrix of the Classifier performance and the generated data with Transferor. **a** The confusion matrix of tissue classification with Classifier. **b** The confusion matrix for the tissue of generated data with Transferor and evaluated by Classifier. **c** The confusion matrix of disease classification with Classifier. (**d**) The confusion matrix for the disease of the generated data with Transferor and evaluated with Classifier

In order to show the *Transferor’s performance* for changing the type of mRNA (normaltumor and tumor normal), we computed its F1-Score (see Table 1), Precision and Recall (Additional file 1: Tables S1 and S2, respectively). To achieve this, we also needed to evaluate the *Classifier performance* with respect to the tissue(breast, prostate, lung, …, etc.) and disease (tumor, normal) beforehand. For this purpose, we report their F1-Score, Precision and Recall, for tissue and disease classification. These performance measures, along with their corresponding confusion matrices summarised in Fig. 2.

**Table 1:** F1 score of tissue classification. (Left), F1 score of tissue classification with Classifier. (Right), F1 score for tissue classification of generated data with Transferor network that evaluated with Classifier

Category	Mean	SD	Category	Mean	SD
Bladder	0.959	0.008	Bladder	0.944	0.032
Breast	0.993	0.001	Breast	0.993	0.004
Cervix	0.935	0.017	Cervix	NaN	NaN
Colon	0.995	0.006	Colon	0.997	0.005
Esophageal	0.999	0.001	Esophageal	0.997	0.003
Kidney	0.992	0.008	Kidney	0.994	0.005
Liver	0.995	0.005	Liver	0.994	0.009
Lung	0.992	0.004	Lung	0.991	0.003
Prostate	0.997	0.005	Prostate	0.994	0.007
Salivary	0.974	0.011	Salivary	0.951	0.035
Stomach	0.998	0.004	Stomach	0.997	0.005
Thyroid	0.999	0.001	Thyroid	0.999	0.001
Uterus	0.959	0.011	Uterus	0.956	0.016

### Performance of classifier compared with other machine learning models

To reduce the data dimensionality, we used principal component analysis (PCA) as a preprocessing step [25]. When dealing with high-dimensional data, it is natural to assume that the latent variables of the data-generating distribution sit on a much lower-dimensional manifold. By finding a lower-dimensional representation through PCA, we preserve important information while removing redundant dimensions, simplifying analysis and modeling. After tuning the parameters of seven traditional machine learning models, we used K-Fold cross-validation with K = 5. we put one-fifth of the data for testing and the other four-fifths for training and validation in each fold. Finally, to report the model's performance, we considered the average performance of the model in different folds. Finally, we compared their performance with performing DeeP4med. Selected baselines are Support vector classifier (SVC), Logistic regression (LR), Linear discriminant analysis (LDA), Naive Bayes (NBayes), Decision tree (DTree), Random forest (RForest), K nearest neighbors (KNN). The results show that DeeP4med has a better performance for identifying tissue types (Additional file 1: Table S3 (left)) and outperforms the other baselines in classifying disease samples (Additional file 1: Table S3 (right)). We should note that the results are consistent using different PCA dimensions (PCA dim = 120, PCA dim = 90, and PCA dim = 150). See Additional file 1: Tables S3, S4, and S5, respectively.

### Biological benchmark

Since our primary purpose was to develop a model that (i) can predict the disease state (i.e., tumor transcriptome) on a patient-by-patient basis based on (RNAseq) healthy tissue information and (ii) predict the healthy state from known tumor information (i.e., RNAseq from tumor biopsies), we designed DeeP4med to produce two types of expression matrices for each tissue:

(1) “transfer tumor” (TT). This data is generated by applying DeeP4med to RNAseq data form normal tissue samples (i.e., original normal data, ON).

For notation clarity, we label this data set as ON_TT

(2) “transfer normal” (TN). This data is generated by applying DeeP4med to RNAseq data from tumor tissue samples (i.e., original tumor data, OT).

For notation clarity, we label this data set as OT_TN.

Half of the data are original in these two types of expression matrices; the remaining are transfers. To evaluate the model's performance of these two matrices in each tissue, (1) DEGs analysis and (2) ENRICHMENT analysis is performed. Then the results were compared against each other. The number of samples in each tissue is shown in Additional file 1: Table S6, and the expression matrices of all tissues are present in Additional file 2: part 1.

### DEG analysis

DEG analysis between tumor and normal states was performed using the limma package [26] on the idep.951 platform [27] between (i) ON and TT groups and (ii) OT and TN groups.

We predicted that if DeeP4med works accurately, there should be a significant overlap of DEGs identified in conditions (i) and (ii).

To test this, genes with adjusted *p*-value ≤ 0.05 and LFC (log fold change, tumor versus normal) ≤ -1 (down-regulated) and LFC ≥ 1 (up-regulated, i.e., when the gene is expressed higher in the tumor compared to the normal) were considered for further analysis. The result files from the idep.951, including DEGs and PCA plots for each tissue, are in the Additional file 2: part 2. We use the Venn diagram tool [28], to identify the intersecting DEG genes (up or downregulated) that are common to conditions (i) and (ii).

Using Eqs. (1) and (2), the true positive rate was calculated (Additional file 1: Table S7).

1 $$True\;positive_{{UP}} = {\text{ }}\frac{{intersect\;(UP)}}{{mean\;UP\;\left( {ON/TT\& OT/TN} \right)}}$$

2 $$True\;positive_{{Down}} = \frac{{intersect\;(Down)}}{{mean\;Down\;\left( {ON/TT\& OT/TN} \right)}}$$

**Fig. 3:**
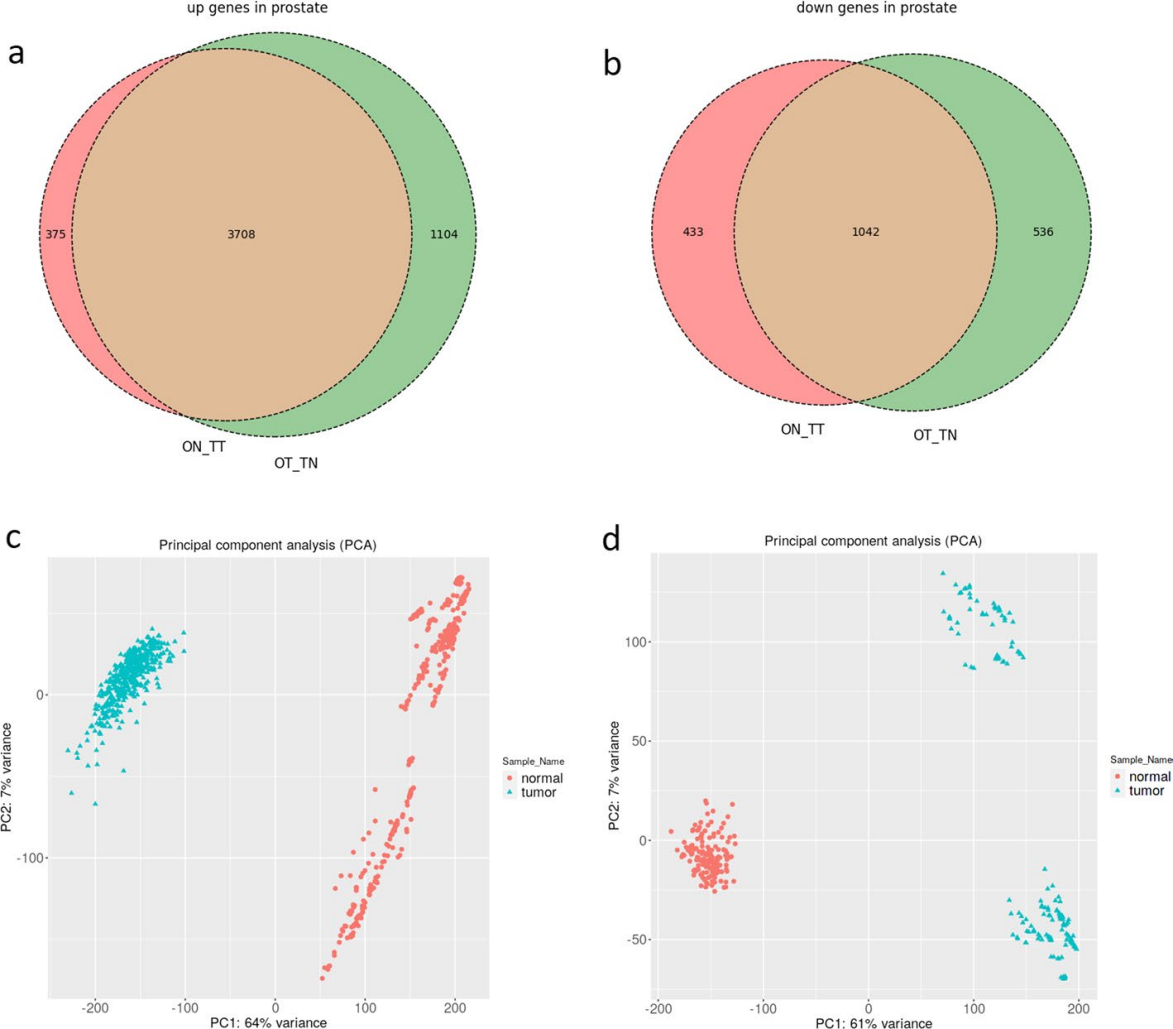
Venn diagram of DEGs and PCA plots. The intersect up (**a**) and down (**b**) genes between OT_TN & ON_TT in the prostate. The PCA plots of prostate samples in_OT_TN (**c**) and ON_TT (**d**) in the prostate. Based on PCA plots, the model could distinguish between tumor and normal samples

The prostate had the highest true positive rate, so we chose it to evaluate the model's performance from a biological aspect. Figure 3 shows the results of the Venn diagram and PCA in the prostate, which shows that the DeeP4med can distinguish between normal and tumor states in each matrix (Venn diagrams of other tissues are in the Additional file 2: part 3).

### Enrichment analysis

By DEGs and using the Enrichment Analysis Visualization Appyter website[29], several enrichment analyzes were performed, which we will discuss below: (1) Gene ontology (GO)_biological process. (2) Cancer cell Line encyclopedia (CCLE) Proteomics. (3) Kyoto encyclopedia of genes and genomes (KEGG) pathway. (4) ChIP enrichment analysis (ChEA). Table 2 shows the results of these four types of enrichment and some examples of intersecting results between the two types of matrices in the 13 types of cancer. Although the enrichment results for all tissues are shown in Table 2, in the following, we will only evaluate the enrichment results of prostate cancer based on the articles. (Results of enrichment analysis for all tissues are presented in the Additional file 2: part 4).

**Table 2:** Enrichment results. Four types of enrichment were performed on ON_TT and OT_TN in the 13 types of cancer. The number of results for each matrix is shown as well as the number of intersecting results. In the last column, the names of some intersecting results are shown

Tissue	Enrichment method	ON_TT output	OT_TN output	Intersect ON_TT & OT_TN	Examples of intersects
Bladder	GO_Biological_Process_2021	557	523	217	Epithelium development (GO:0060429) MAPK cascade (GO:0000165) Ras protein signal transduction (GO:0007265)
	CCLE_Proteomics_2020	5	4	3	T24 URINARY TRACT TenPx37 UBLC1 URINARY TRACT TenPx15 KU1919 URINARY TRACT TenPx36
	KEGG 2021 Human	46	23	13	Pathways in cancer TGF-beta signaling pathway Cell cycle
	ChEA_2016	14	12	4	FOXP1 21,924,763 ChIP-Seq HESCs Human RELA 24523406 ChIP-Seq FIBROSARCOMA Human FOXP3 21,729,870 ChIP-Seq TREG Human CTNNB1 24,651,522 ChIP-Seq LGR5 + INTESTINAL STEM Human
Breast	GO_Biological_Process_2021	498	602	265	Mammary gland epithelium development (GO:0061180) Actin filament organization (GO:0007015) Regulation of mitotic cell cycle phase transition (GO:1,901,990)
	CCLE_Proteomics_2020	7	9	4	HCC1395 BREAST TenPx15 MDAMB453 BREAST TenPx19 HCC1187 BREAST TenPx20 HCC2218 BREAST TenPx21
	KEGG 2021 Human	31	25	14	FOXO signaling pathway TGF-beta signaling pathway Ras signaling pathway
	ChEA_2016	6	8	4	FOXP1 21,924,763 ChIP-Seq HESCs Human TFEB 21752829 ChIP-Seq HELA Human FOXA1 26,769,127 Chip-Seq PDAC-Cell line Human FOXA2 19,822,575 ChIP-Seq HepG2 Human
Cervix	GO_Biological_Process_2021	491	574	189	Negative regulation of Wnt signaling pathway (GO:0090090) Regulation of mitotic cell cycle phase transition (GO:1,901,990) Regulation of transcription by RNA polymerase II (GO:0006357)
	CCLE_Proteomics_2020	0	0	0	–
	KEGG 2021 Human	37	49	18	Human papillomavirus infection Pathways in cancer Hedgehog signaling pathway IL-17 signaling pathway
	ChEA_2016	12	6	4	SUZ12 18,692,474 ChIP-Seq MEFs Mouse VDR 23401126 ChIP-Seq LCL-AND-THP1 Human RELA 24523406 ChIP-Seq FIBROSARCOMA Human CTNNB1 24,651,522 ChIP-Seq LGR5 + INTESTINAL STEM Human
Colon	GO_Biological_Process_2021	499	469	193	Rho protein signal transduction (GO:0007266) Epithelium development (GO:0060429) TNF-mediated signaling pathway (GO:0033209)
	CCLE_Proteomics_2020	13	13	8	SNUC1 LARGE INTESTINE TenPx19 HCC56 LARGE INTESTINE TenPx07 SW948 LARGE INTESTINE TenPx11 RKO LARGE INTESTINE TenPx04
	KEGG 2021 Human	29	37	13	Hedgehog signaling pathway Pathways in cancer Cell cycle Ras signaling pathway
	ChEA_2016	9	12	6	FOXP1 21,924,763 ChIP-Seq HESCs Human RELA 24523406 ChIP-Seq FIBROSARCOMA Human HNF4A 19822575 ChIP-Seq HepG2 Human KDM2B 26808549 Chip-Seq K562 Human
Esophageal	GO_Biological_Process_2021	586	582	278	MAPK cascade (GO:0000165) Cellular protein catabolic process (GO:0044257) Epithelium development (GO:0060429) Regulation of EGFR signaling pathway (GO:0042058)
	CCLE_Proteomics_2020	4	9	2	TE4 OESOPHAGUS TenPx33 KYSE410 OESOPHAGUS TenPx38
	KEGG 2021 Human	59	41	27	Pathways in cancer TGF-beta signaling pathway Hedgehog signaling pathway mTOR signaling pathway PI3K-Akt signaling pathway
	ChEA_2016	11	10	7	KDM2B 26808549 Chip-Seq K562 Human FOXP1 21,924,763 ChIP-Seq HESCs Human VDR 23401126 ChIP-Seq LCL-AND-THP1 Human HNF4A 19822575 ChIP-Seq HepG2 Human
Kidney	GO_Biological_Process_2021	613	515	250	Regulation of immune response (GO:0050776) Kidney development (GO:0001822) TNF-mediated signaling pathway (GO:0033209)
	CCLE_Proteomics_2020	6	5	4	A498 KIDNEY TenPx05 CAKI1 KIDNEY TenPx36 769P KIDNEY TenPx25 OSRC2 KIDNEY TenPx20
	KEGG 2021 Human	50	39	23	Pathways in cancer TNF signaling pathway Colorectal cancer Cellular senescence
	ChEA_2016	6	17	4	RELA 24523406 ChIP-Seq FIBROSARCOMA Human FOXP1 21,924,763 ChIP-Seq HESCs Human KDM2B 26808549 Chip-Seq K562 Human PRDM5 23,873,026 ChIP-Seq MEFs Mouse
Liver	GO_Biological_Process_2021	835	581	340	ERK1 and ERK2 cascade (GO:0070371) Intrinsic apoptotic signaling pathway (GO:0097193) Programmed necrotic cell death (GO:0097300)
	CCLE_Proteomics_2020	9	9	8	JHH1 LIVER TenPx34 HEP3B217 LIVER TenPx02 JHH7 LIVER TenPx05 HEPG2 LIVER TenPx02
	KEGG 2021 Human	77	63	43	FoxO signaling pathway MAPK signaling pathway NF-kappa B signaling pathway Ras signaling pathway
	ChEA_2016	13	9	7	TP63 19,390,658 ChIP-ChIP HaCaT Human HNF4A 19822575 ChIP-Seq HepG2 Human FOXP1 21,924,763 ChIP-Seq HESCs Human VDR 23401126 ChIP-Seq LCL-AND-THP1 Human
Lung	GO_Biological_Process_2021	508	453	196	Protein processing (GO:0016485) Regulation of RNA metabolic process (GO:0051252) NIK/NF-kappaB signaling (GO:0038061)
	CCLE_Proteomics_2020	38	29	23	LU65 LUNG TenPx22 DMS273 LUNG TenPx06 DV90 LUNG TenPx12 LUDLU1 LUNG TenPx09
	KEGG 2021 Human	22	32	11	Sphingolipid metabolism Pathways in cancer Cellular senescence Focal adhesion
	ChEA_2016	4	10	4	RELA 24523406 ChIP-Seq FIBROSARCOMA Human FOXP1 21,924,763 ChIP-Seq HESCs Human CTNNB1 24,651,522 ChIP-Seq LGR5 + INTESTINAL STEM Human GABP 19822575 ChIP-Seq HepG2 Human
Prostate	GO_Biological_Process_2021	487	455	264	MAPK cascade (GO:0000165) regulation of EGFR signaling pathway (GO:0042058) cellular response to FGF stimulus (GO:0044344) Fc receptor signaling pathway (GO:0038093)
	CCLE_Proteomics_2020	3	1	1	VCAP PROSTATE TenPx21
	KEGG 2021 Human	40	36	21	Ras signaling pathway Pathways in cancer Regulation of actin cytoskeleton Rap1 signalling pathway
	ChEA_2016	3	13	3	FOXP1 21,924,763 ChIP-Seq HESCs Human RELA 24523406 ChIP-Seq FIBROSARCOMA Human KDM2B 26808549 Chip-Seq K562 Human
Salivary	GO_Biological_Process_2021	505	464	173	Gland morphogenesis (GO:0022612) Positive regulation of secretion by cell (GO:1,903,532) Wound healing (GO:0042060) Polarized epithelial cell differentiation (GO:0030859)
	CCLE_Proteomics_2020	0	0	0	–
	KEGG 2021 Human	29	40	13	Protein processing in endoplasmic reticulum Protein export Ras signaling pathway Glycerolipid metabolism Focal adhesion
	ChEA_2016	9	6	2	RELA 24523406 ChIP-Seq FIBROSARCOMA Human ESR1 21,235,772 ChIP-Seq MCF-7 Human
Stomach	GO_Biological_Process_2021	430	474	219	Polarized epithelial cell differentiation (GO:0030859) NIK/NF-kappaB signaling (GO:0038061) VEGFR signaling pathway (GO:0048010)
	CCLE_Proteomics_2020	2	5	2	HGC27 STOMACH TenPx06 KATOIII STOMACH TenPx15
	KEGG 2021 Human	36	26	14	Ubiquitin mediated proteolysis Adipocytokine signaling pathway TNF signaling pathway AMPK signaling pathway JAK-STAT signaling pathway
	ChEA_2016	11	15	9	FOXP1 21,924,763 ChIP-Seq HESCs Human CTNNB1 24,651,522 ChIP-Seq LGR5 + INTESTINAL STEM Human TFEB 21752829 ChIP-Seq HELA Human KDM2B 26808549 Chip-Seq K562 Human HNF4A 19822575 ChIP-Seq HepG2 Human
Thyroid	GO_Biological_Process_2021	434	497	226	Recombinational repair (GO:0000725) Protein polyubiquitination (GO:0000209) Interleukin-1-mediated signaling pathway (GO:0070498)
	CCLE_Proteomics_2020	2	1	1	8305C THYROID TenPx30
	KEGG 2021 Human	25	36	12	Calcium signaling pathway Pathways in cancer TGF-beta signaling pathway IL-17 signaling pathway Regulation of actin cytoskeleton
	ChEA_2016	8	13	2	RELA 24523406 ChIP-Seq FIBROSARCOMA Human KDM2B 26808549 Chip-Seq K562 Human
Uterus	GO_Biological_Process_2021	566	499	255	Cytokine-mediated signaling pathway (GO:0019221) MAPK cascade (GO:0000165) Rho protein signal transduction (GO:0007266) Negative regulation of Wnt signaling pathway (GO:0030178)
	CCLE_Proteomics_2020	7	8	5	HEC108 ENDOMETRIUM TenPx39 JHUEM2 ENDOMETRIUM TenPx28 HEC59 ENDOMETRIUM TenPx25 HEC265 ENDOMETRIUM TenPx37 SNU685 ENDOMETRIUM TenPx33
	KEGG 2021 Human	51	48	23	Pathways in cancer Human papillomavirus infection Regulation of actin cytoskeleton Basal cell carcinoma MAPK signaling pathway PI3K-Akt signaling pathway
	ChEA_2016	12	8	4	RELA 24523406 ChIP-Seq FIBROSARCOMA Human FOXP1 21,924,763 ChIP-Seq HESCs Human XRN2 22,483,619 ChIP-Seq HELA Huma

#### CCLE_Proteomics_2020 enrichment analysis

According to Table 2, VCAP is present in the enrichment result of both types of prostate matrix. Using the TCGA-110CL (https://comphealth.ucsf.edu/app/tcga-110) website, the expression profiles of real prostate cancer samples in the TCGA database were compared with the expression profiles of different cell lines, as shown in the Additional file 1: Fig. S1. This figure shows that the VCAP cell line correlates most with prostate cancer samples. Therefore, the nature of the data produced by DeeP4med is consistent with real data. The number of cell lines that have been correctly identified by enrichment analysis for each tissue and the best cell line and its P-value are shown in the Additional file 1: Table S8. The expression matrix generated by the model was correctly identified in 11 of the 13 tissues. Only salivary and cervical tissues lacked the appropriate cell lines. However, for salivary, five cell lines such as BICR6, SCC25, HSC4, BICR22, and CAL27, were identified that were anatomically close to this tissue (Additional file 2: part 4_salivary section).

#### KEGG 2021 human enrichment analysis

According to Table 2, there are 21 intersect metabolic pathways in the prostate, and the Ras signaling pathway is one of the most important of them, so we discuss its role in prostate cancer (a complete list of metabolic pathways is shown in the Additional file 2: part 4_prostate section). In 2009, Pearson et al. [30] showed malfunction in Wnt and Ras signaling, and mutations in K-ras and beta-catenin can lead to invasive carcinoma in the prostate. In 2016, Chen et al. [31] by text mining the prostate cancer articles, extracted 41 important proteins, and created a protein–protein interaction (PPI) network. By applying functional annotation on a network, they find Ras protein signal transduction is one of the important signaling pathways in prostate cancer. Also in 2021, Strittmatter et al. [32] show the change in ERG expression gene by Ras/ERK and PI3K/AKT signaling pathways, promoting prostate tumor.

#### GO_Biological_Process_2021 analysis

Based on enrichment results in Table 2, 264 intersect biological processes in prostate cancer were obtained (In the Additional file 2: part 4_prostate section). The MAPK cascade intersects between two matrices and is a key downstream of Ras signaling, so we choose the MAPK cascade to discuss its role in prostate cancer. A search of the coremine (https://www.coremine.com/medical/#search) database revealed that there were approximately 20 articles related to prostate cancer and MAPK cascade (GO:0000165) and 12 articles related to actin cytoskeleton reorganization (GO:0031532) and prostate cancer. In 2019, Wu et al. [33] with an analysis of expression and methylation profiles of prostate cancer, find 322 genes that were hypermethylated and downregulated. By enriching these genes, they found one of the important biological processes was the MAPK cascade. In 2020, Singh et al. [34] for the identification of biomarkers in prostate cancer, analysis proteomics profile of prostate cancer cell lines such as LNCaP and PC-3 by mass spectrometry, they found 474 proteins were deregulated. Enrichment analysis reveals that some biological processes, such as the MAPK cascade, have an essential role in the initiation and progression of cancer. In 2021, Shen et al. [35] MAPK4 expression (one gene of MAPK cascade) promoted prostate cancer cell proliferation, so this gene was a potential target for prostate cancer treatment.

#### ChEA_2016 enrichment analysis

According to Table 2, FOXP1, RELA, and KDM2B, transcription factors intersect in OT_TN & ON_TT in prostate cancer. The Coremine website finds approximately 18, 250, and 4 articles for FOXP1, RELA, and KDM2B related to prostate cancer. In 2021, Panigrahi et al. [36] knocked down the RAD9 gene in prostate cancer DU145 cells and found that expression of FOXP1 were down-regulated, so migration and proliferation of tumor cell decreased. In 2022, Raspin et al. [37] investigate some gene fusions in prostate cancer in TCGA data. One of the genes fusion related to RYBP: FOXP1. (complete list of transcription factors enrichment is shown in the Additional file 2: part 4_prostate section).

To better evaluate the performance of our model, we compared the matrices produced in the model with the original matrix (ON/OT). We used two different approaches: a biological approach using the DEGs method and a statistical approach using the PCA and UMAP methods which show the distribution of samples by reducing the dimensions. The output of our model for each tissue is two types of matrices: OT/TN and ON/TT. Next, we separately compared the DEGs obtained from each of these matrices with the DEGs obtained from the true matrix by the Venn diagram. There are 13 tissues; for each tissue, there are two comparisons (OT/TN vs. original and ON/TT vs. original), and in each comparison, two states of up and down were analyzed separately. Therefore, 52 Venn diagrams were obtained (Additional file 2: part 5_Venny and DEGs). The results show that depending on the type of tissue, a sufficient number of DEGs (UP & Down genes) are common in these three types of matrices, which indicates that our model has been able to produce matrices that are similar to the true matrix. Also, PCA and UMAP plots show that in three types of matrices (original, OT/TN, and ON/TT), normal samples' distribution differs from tumor samples. These results indicate that our model has understood the pattern of normal and tumor samples and produced new matrices (Additional file 2: part 5_PCA&UMAP). Also, the list of common DEGs between all three matrices and their Venn diagram in Additional file 2: part 6 is available. These genes are the most important because they exist in all three matrices.

## Discussion

By focusing on each patient and understanding the complex molecular mechanisms of the disease and its interaction with environmental factors and individual genetic diversity, P4 medicine has become the most effective approach in personalized medicine. By applying system biology methods, P4 medicine's primary goal is to make the disease state predictable, preventable, and curable. However, individuals' genetic and demographic information affects the molecular mechanisms that drive the disease stage, and identifying them requires deep learning approaches. In this work, we developed a transfer model capable of predicting the disease state using RNAseq data (i.e., bulk transcriptomics). Transcriptomic data is readily available through different projects (i.e., TCGA) and is also more dynamic than genomic data alone, as it also reveals changes in the epigenome of cells and how gene expression is modulated by different disease conditions but also, in the context of cancer cells, by the interaction of tumor cells and the tumor microenvironment. That means RNAseq captures the changes in disease cells by measuring the cell's gene expression profile. Because the changes in all genes are measured, RNAseq data is very comprehensive and suitable for applications in Deep Learning. Our fundamental goal in developing DeeP4med was to use deep learning to predict changes in gene expression profiles. In this regard, previous work has attempted to do this using different datasets. For example, DeepChrome uses histone modifications to predict gene expression profiles [38]. HE2RNA use histopathology images to predict gene expression profiles in tumor [39] or tuberculosis [40]. Some models, like Enformer [41] or similar models [42, 43], predict gene expression from DNA sequences. DeeP4med predicts normal gene expression from tumor gene expression and vice versa. One of DeeP4med's uses is to predict how cancer would look if happening to a normal person. Suppose we have a normal gene expression profile of a healthy person in a specific tissue. The model can predict the probable tumor profile of that healthy person in the future. So we can find out which genes are involved in this process and reduce the risk of cancer in that person by prescribing certain drugs or taking special care. By developing such models using other omics data such as genomics, proteomic or metabolic, we can hope that besides predicting the expression profile, the model can also suggest specific and proper drugs for treatment. Developing this model and its capacity to predict the tumor state from healthy conditions will stimulate further research in P4 medicine. One of the therapeutic aspects of developing such models is integrating them with models that use deep learning in drug Repositioning [44, 45]. The use of combined models is a new horizon in the diagnosis and treatment of diseases.

## Method

Our deep learning model contains two separate deep models, Classifier and Transferor, based on their function. We first trained a neural network called Classifier to classify the type of gene expression profiles (tumor or normal) and their corresponding tissues. Then, using Classifier as our discriminator, we trained Transferor, an autoencoder for transferring the type of gene expressions from the tumor to their nearest normal version and vice versa while keeping their tissue of origin unchanged. The Transferor is conditioned on the type of input sample (tumor or normal) and simultaneously generates the normal and tumoral versions of the input. Classifier helps us accurately measure the performance of the Transferor in terms of concordance between the expected type and tissue and that of the first generated mRNA. Another performance measure that has been introduced to the loss function of the Transferor is the mean squared distance between the input sample and the second generated mRNA.

### Experimental setup

The loss function of the Transferor is a weighted sum of three losses. The first two losses are computed based on the Classifier's output and measure the Transferor's performance as a classification task. The third loss computes the distance between the generated mRNA and the input and can be considered a regression task. We used mean squared error to measure the distance between the input and the generated mRNA. We use cosine similarity to measure the correspondence between the type and tissue of the input and generated mRNA [46]. To assess the performance of the model, we used fivefold cross-validation.

### Classifier

**Fig. 4:**
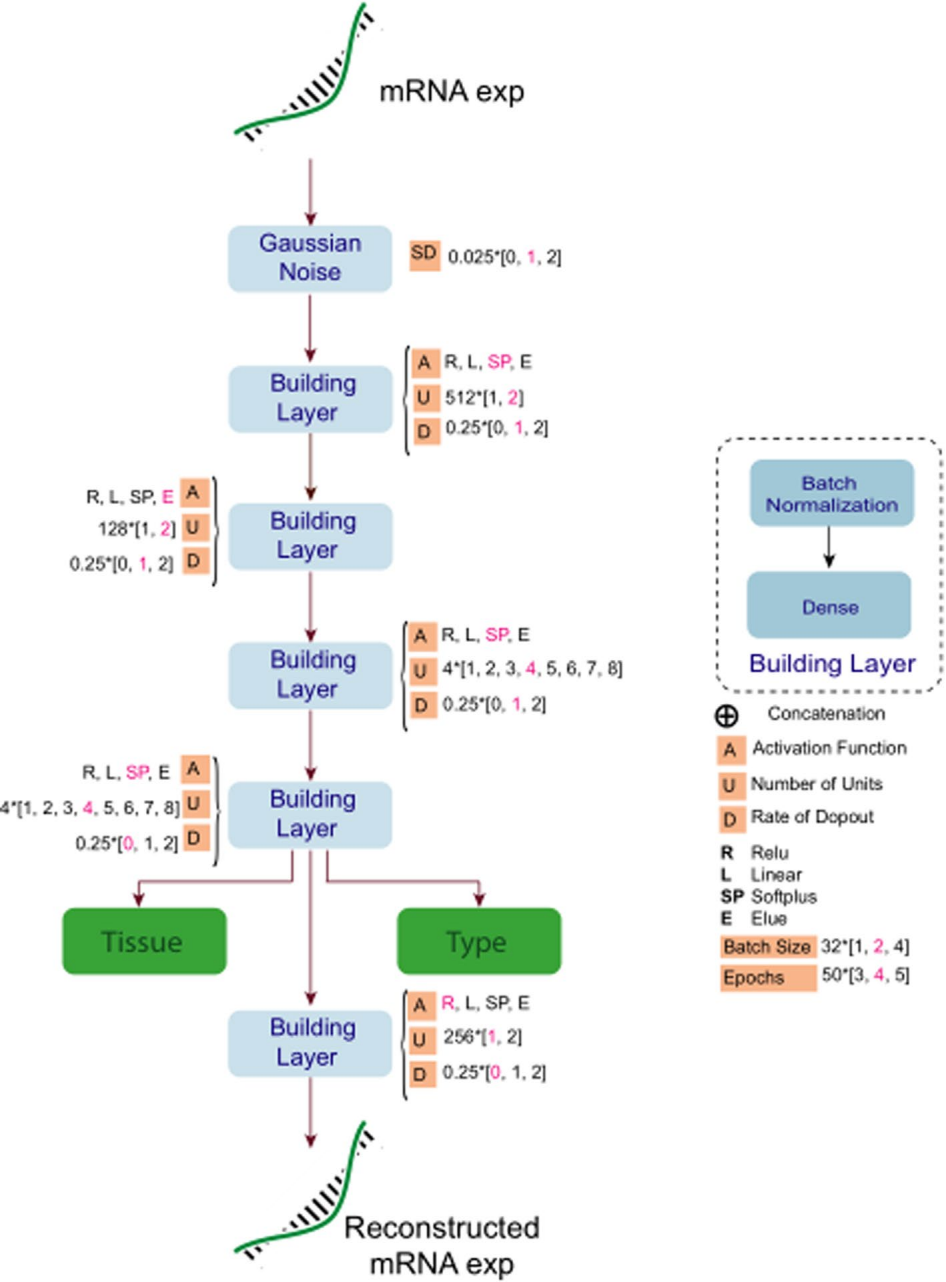
The architecture of Classifier. This model gets an mRNA expression matrix as input and has three outputs, including tissue and type, modelled as a classification task, and mRNA expression, modelled as a regression task. The total loss function for training this network is a weighted sum of three losses (which are the cosine similarity between the predicted tissue and ground truth tissue, the cosine similarity between the predicted type and ground truth type, and the mean square error between the input mRNA expression and the reconstructed mRNA)

The classifier has an architecture similar to that of the model proposed in DeePathology [46], which is an autoencoder augmented with two classifiers (see Fig. 4). In this work, after training the whole proposed architecture, we remove all layers related to mRNA reconstruction and only use the type and tissue classifier layers. Following DeePathology [46], to show that our autoencoder effectively separates the input samples, we visualize the embeddings at the bottleneck layer of DeeP4med.

This network can be symbolized as:

3 $$({tissue}_{output},{type}_{output},{mRNA}_{output})={MLP}_{\gamma }^{autoencoder}({mRNA}_{input})$$

Combination of autoencoder and classifier with weighted loss as below:

4 $$\begin{aligned} & w_{1} *MSE\left( {mRNA_{{input}} ,mRNA_{{output}} } \right) + w_{2} *Cosine\;Distance\left( {type_{{input}} ,type_{{output}} } \right) \\ & + w_{3} *Cosine\;Distance\left( {tissue_{{input}} ,tissue_{{output}} } \right) \\ \end{aligned}$$

Such that ${w}_{1},{w}_{2}$ and ${w}_{3}$ are weights and we set as they have used before [46]. We used only the classifier part of this network in learning Transferor, as we explained in Eq. (8).

### Transferor

**Fig. 5:**
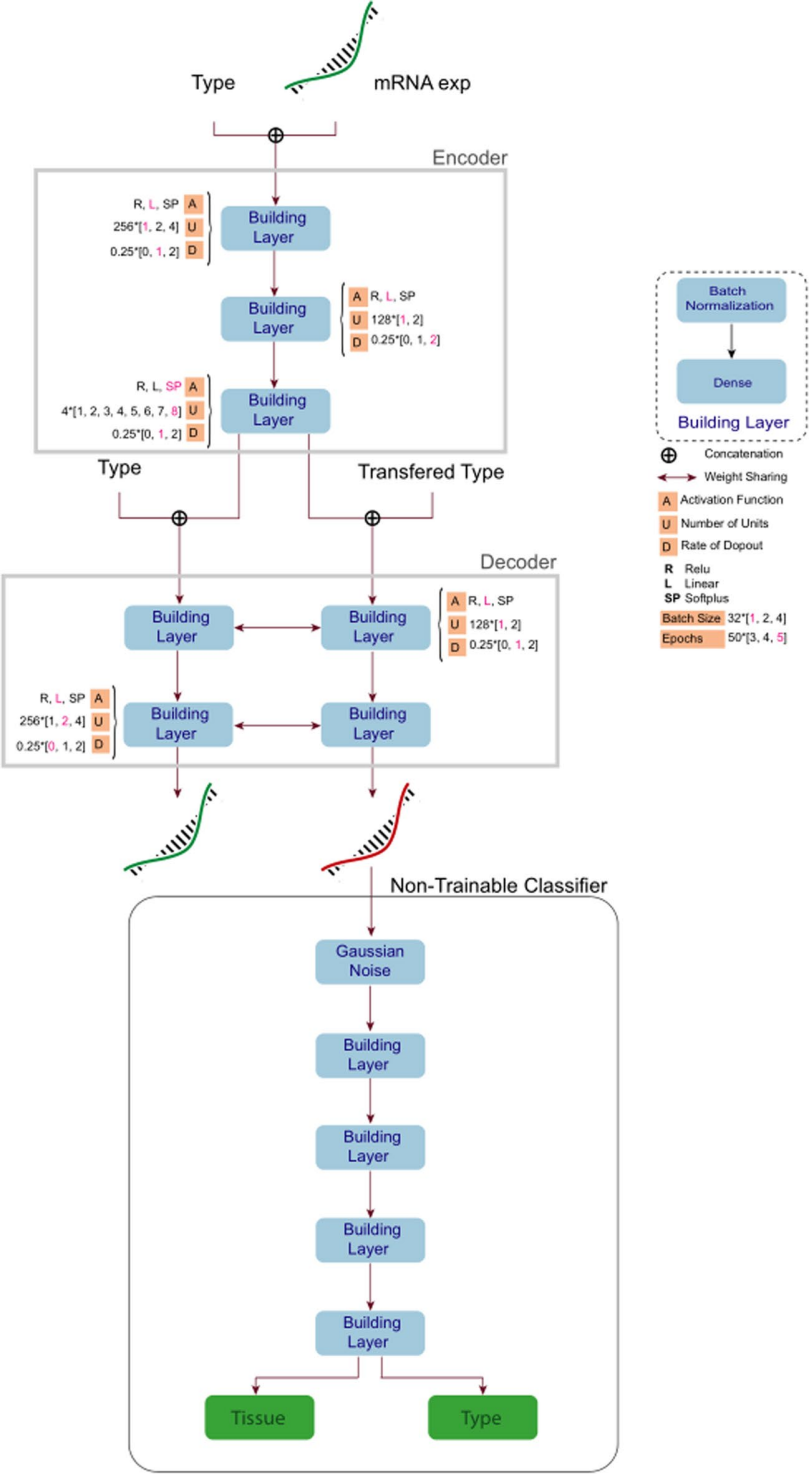
The high-level architecture of Transferor together with Classifier for transferring mRNA profile. One output of the Transferor is fed to the Classifier to measure its performance. Transferor gets mRNA and its type as inputs of the encoder and embeds these two inputs to the last layer of the encoder. Then, the embedded vector is given once with its original type and once with the transferred type (opposite of its original type) to the decoder as inputs. The mean square error of this output and input mRNA expression is included as a part of the total loss of the Transferor. The total loss also includes the cosine similarity between predicted tissue with Classifier and ground truth tissue of input mRNA and the cosine similarity between the predicted type with Classifier and transferred type. Hence, the total loss function of the Transferor is a weighted sum of three losses

The transferor consists of an encoder and two decoders that share parameters. In each forward pass of the model, an mRNA profile and its type are encoded and again concatenated to each type separately. The resulting vectors are encoded sequences of mRNA concatenated with a type that is opposite or the same as the input. Then, the type of augmented embeddings is fed to the decoders individually (Fig. 5). Formally, we can summarise the encoding process in Eq. (5).

5 $$h={MLP}_{\phi }^{enc}({mRNA}_{input};type)$$

MLPφ shows our encoder is parametrized by φ and h is the embedding of an mRNA given its type. Equations (6) and (7) show decoding.

6 $${mRNA}_{dec}^{(1)}={MLP}_{\psi }^{dec}(h;{type}_{original})$$

7 $${mRNA}_{dec}^{(2)}={MLP}_{\psi }^{dec}(h;{type}_{opposite})$$

We want DeeP4med to keep the tissue unchanged but control the type of mRNA. Formally, it should satisfy Eq. (8):

8 $$({type}_{output};{tissue}_{output})={MLP}_{\theta }^{Classifier}({mRNA}_{dec}^{(2)})$$

Each output of the Transferor contributes to the loss function: The first output, which should have the same tissue but the opposite type as the input, is evaluated by the Classifier. The second output, which should have the same tissue and the same type as the input, is used to measure the similarity between the input and output. Finally, the total loss for this network is a weighted sum of the cosine distance between the Classifier’s outputs and the expected tissue and type and the mean squared distance between the generated mRNA and the input. Mathematically, we have Eq. (9):

9 $$\begin{aligned} Loss_{{Total}} & = w_{1} \cdot MSE\left( {mRNA_{{dec}}^{{\left( 1 \right)}} ;mRNA_{{input}} } \right) \\ & \quad + w_{2} \cdot Cosine\,Distance\left( {type_{{opposite}} ;type_{{output}} } \right) \\ & \quad + w_{3} \cdot Cosine\,Distance\left( {tissue_{{input}} ;tissue_{{output}} } \right) \\ \end{aligned}$$

At this stage, only the Transferor parameters are updated, and the classifier parameters are frozen.

### Hyperparameter tuning

In Neural Networks (NN), there are many hyperparameters, and tuning them is critical for finding the best model. Given that we used multilayer perceptron networks, the hyperparameters are:

- *Units*: The number of neurons in layers is critical to finding the best architecture.
- *Activation function*: In Artificial neural network (ANN), an activation function is applied after a weighted sum of input for each neuron. ReLU (Rectified linear unit) [47], defined as f(x) = max(0; x), widely used in ANN, was one of our selections for the activation function. Softplus (f(x) = ln(1 + e^x^))[48] and Linear (f(x) = x) are another of our selection. Also, we use Elu (Exponential linear unit), which is defined as Eq. (10):
  10 $$f(x) = \left\{ {\begin{array}{*{20}l} {e^{x} - 1 \le 0} \hfill \\ {x > 0} \hfill \\ \end{array} } \right.$$
- *Dropout rate*: Dropout layer set to zero values with probability as defined rate. This is a widely used technique for preventing overfitting in recent years.

The hyperparameter search space is shown in the Additional file 1: Tables S9 and S10.

## Conclusion

A general review of all the results shows that DeeP4med has been successful in terms of machine learning methods. Also, regarding biological results, DeeP4med performs relatively well depending on the tissue type. Of course, DeeP4medis still needs to complete and have considered all aspects. Indeed, the performance of the model can be improved in future studies.

## Supplementary Information

**Additional file 1**. Details of computational performance and hyperparameter space of the DeeP4med (Transferor and Classifier) as well as biological evaluation of the model.

**Additional file 2.** Dataset (gene expression matrix of different tissues), DEGs and enrichment analysis results, common and important genes between different matrices.

## Data Availability

The datasets analysed during the current study and its supplementary information files are available in the google drive repository, (https://drive.google.com/drive/folders/1lMMQdMXsHT8fcP9Mz9sb6NpyFByI7rcj?usp=share_link). The code is available from the corresponding author upon reasonable request (stahmasebian@gmail.com).

## Abbreviations

DEGs: Differentially expressed genes
TCGA: The Cancer Genome Atlas
GTEx: Genotype-tissue expression
SVC: Support vector classifier
LR: Logistic regression
LDA: Linear discriminant analysis
NBayes: Naive Bayes
DTree: Decision tree
RForest: Random forest
KNN: K nearest neighbors
PCA: Principal component analysis
TT: Transfer tumor
ON: Original normal
TN: Transfer normal
OT: Original tumor
LFC: Log fold change
GO: Gene ontology
CCLE: Cancer cell line encyclopedia
KEGG: Kyoto encyclopedia of genes and genomes
ChEA: ChIP enrichment analysis
PPI: Protein–protein interaction
ReLU: Rectified linear unit
Elu: Exponential linear unit
MLPM: Machine learning for personalized medicine
ANN: Artificial neural network
