# Supplementary material for: DeeP4med: deep learning for P4 medicine to predict normal and cancer transcriptome in multiple human tissues
Source: BMC Bioinformatics. 2023 Jul 4;24:275. doi: 10.1186/s12859-023-05400-2 (PMC10320882; doi:10.1186/s12859-023-05400-2)
Supplement: Supplementary file 1 — Additional file 1. Details of computational performance and hyperparameter space of the DeeP4med (Transferor and Classifier) as well as biological evaluation of the model. [file 12859_2023_5400_MOESM1_ESM.docx]

**DeeP4med: deep learning for P4 medicine to predict normal and cancer transcriptome in multiple human tissues**

**Roohallah Mahdi Esferizi^1,+^, Behnaz Haji Molla Hoseyni^2,+^, Amir Mehrpanah^3^, Yazdan Golzade^4^, Ali Najafi^5^, Fatemeh Elahian^1^, Amin Zadeh Shirazi^6^, Guillermo A. Gomez^6^, and Shahram Tahmasebian^7,^***

^1^ Department of Medical Biotechnology, School of Advanced Technologies, Shahrekord University of
Medical Sciences, Shahrekord, Iran ([roohallah1435@gmail.com](mailto:roohallah1435@gmail.com), [dr.elahian@yahoo.com](mailto:dr.elahian@yahoo.com))
^2^ Laboratory of Systems Biology and Bioinformatics (LBB), University of Tehran, Tehran, Iran
([hoseyni.sb@gmail.com](mailto:hoseyni.sb@gmail.com))
^3^ Faculty of Mathematics, Shahid Beheshti University, Tehran, Iran ([a.mehrpanah@mail.sbu.ac.ir](mailto:a.mehrpanah@mail.sbu.ac.ir))
^4^ Department of Mathematics, Faculty of Basic Sciences, Iran University of Science and
Technology,(IUST). Tehran, Iran. ([Yazdan.golzade@gmail.com](mailto:Yazdan.golzade@gmail.com))
^5^ Molecular Biology Research Center, Systems Biology and Poisonings Institute, Baqiyatallah University of Medical Sciences, Tehran, Iran. ([najafi74@bmsu.ac.ir](mailto:najafi74@bmsu.ac.ir))
^6^ Centre for Cancer Biology, SA Pathology and University of South Australia, Adelaide, SA 5000,
Australia ([amin.zadeh_shirazi@mymail.unisa.edu.au](mailto:amin.zadeh_shirazi@mymail.unisa.edu.au), [Guillermo.Gomez@unisa.edu.au](mailto:Guillermo.Gomez@unisa.edu.au))
^7^ Cellular and Molecular Research Center, Basic Health Sciences Institute, Shahrekord University of
Medical Sciences, Shahrekord, Iran ([stahmasebian@gmail.com](mailto:stahmasebian@gmail.com))
+ Authors Contributed Equally
 * Corresponding author

**Additional file 1:** **Figure S1 | Correlation between different prostate cancer cell lines with *TCGA* samples*.*** based on transcriptomic analysis by the TCGA-110CL website, VCAP cell line is most correlated with actual prostate cancer samples. This cell line was correctly predicted by enriching the data generated by the DeeP4med.


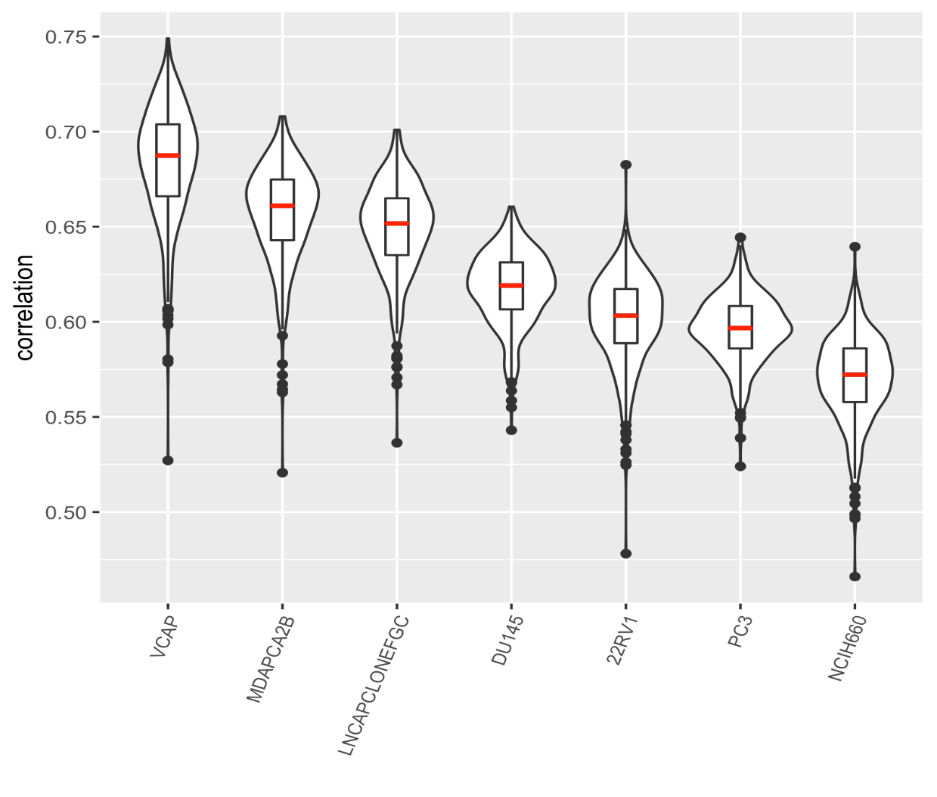


**Additional file 1:** **Table S1 | Precision of tissue classification.** (**left**) precision of tissue classification with Classifier. (**right**) precision for tissue classification of generated data with Transferor network that evaluated with Classifier.

| **Category** | **Mean** | **SD** | **Category** | **Mean** | **SD** |
| --- | --- | --- | --- | --- | --- |
| Bladder | 0.962 | 0.024 | Bladder | 0.926 | 0.055 |
| Breast | 0.992 | 0.004 | Breast | 0.992 | 0.008 |
| Cervix | 0.941 | 0.032 | Cervix | NaN | NaN |
| Colon | 0.994 | 0.005 | Colon | 0.996 | 0.005 |
| Esophageal | 1.000 | 0.000 | Esophageal | 0.996 | 0.006 |
| Kidney | 0.993 | 0.008 | Kidney | 0.998 | 0.003 |
| Liver | 0.998 | 0.004 | Liver | 0.993 | 0.013 |
| Lung | 0.994 | 0.004 | Lung | 0.987 | 0.004 |
| Prostate | 0.996 | 0.007 | Prostate | 0.988 | 0.013 |
| Salivary | 0.970 | 0.017 | Salivary | 0.938 | 0.071 |
| Stomach | 0.996 | 0.007 | Stomach | 1.000 | 0.000 |
| Thyroid | 0.999 | 0.002 | Thyroid | 0.999 | 0.002 |
| Uterus | 0.953 | 0.010 | Uterus | 0.937 | 0.039 |

**Additional file 1: Table S2 | Recall of tissue classification.** (**left**) recall of tissue classification with classifier. (**right**) recall for tissue classification of generated data with Transferor network that evaluated with Classifier.

| **Category** | **Mean** | **SD** | **Category** | **Mean** | **SD** |
| --- | --- | --- | --- | --- | --- |
| Bladder | 0.957 | 0.015 | Bladder | 0.964 | 0.014 |
| Breast | 0.993 | 0.003 | Breast | 0.994 | 0.003 |
| Cervix | 0.929 | 0.013 | Cervix | 0.714 | 0.357 |
| Colon | 0.996 | 0.007 | Colon | 0.998 | 0.005 |
| Esophageal | 0.999 | 0.002 | Esophageal | 0.999 | 0.002 |
| Kidney | 0.991 | 0.008 | Kidney | 0.991 | 0.008 |
| Liver | 0.991 | 0.008 | Liver | 0.996 | 0.005 |
| Lung | 0.990 | 0.006 | Lung | 0.994 | 0.003 |
| Prostate | 0.998 | 0.004 | Prostate | 1.000 | 0.000 |
| Salivary | 0.978 | 0.012 | Salivary | 0.968 | 0.019 |
| Stomach | 1.000 | 0.000 | Stomach | 0.993 | 0.010 |
| Thyroid | 1.000 | 0.000 | Thyroid | 1.000 | 0.000 |
| Uterus | 0.966 | 0.021 | Uterus | 0.977 | 0.020 |

**Additional file 1:** **Table S3 | Accuracy of different machine learning algorithms and our deep-learning-based approach by PCA dim = 120.** (left) accuracy on the task of tissue type identification. (right) accuracy on the task of disease-normal classification.

| Disease classification accuracy (PCA dim=120) | | | | |  | Tissue classification accuracy (PCA dim = 120) | | | | | |
| --- | --- | --- | --- | --- | --- | --- | --- | --- | --- | --- | --- |
| **max** | **median** | **min** | **std** | **mean** |  | **max** | **median** | **min** | **std** | **mean** | **Model** |
| 0.994 | 0.992 | 0.989 | 0.002 | 0.992 |  | 0.988 | 0.986 | 0.985 | 0.002 | 0.986 | Ours |
| 0.987 | 0.981 | 0.977 | 0.004 | 0.982 |  | 0.984 | 0.982 | 0.98 | 0.002 | 0.982 | SVC |
| 0.988 | 0.981 | 0.98 | 0.003 | 0.982 |  | 0.985 | 0.982 | 0.979 | 0.002 | 0.982 | LR |
| 0.982 | 0.97 | 0.958 | 0.009 | 0.97 |  | 0.976 | 0.97 | 0.958 | 0.007 | 0.968 | LDA |
| 0.893 | 0.883 | 0.866 | 0.01 | 0.882 |  | 0.896 | 0.885 | 0.881 | 0.006 | 0.888 | NBayes |
| 0.95 | 0.855 | 0.848 | 0.044 | 0.872 |  | 0.866 | 0.858 | 0.848 | 0.008 | 0.858 | DTree |
| 0.971 | 0.945 | 0.936 | 0.013 | 0.95 |  | 0.951 | 0.947 | 0.94 | 0.005 | 0.946 | RForest |
| 0.984 | 0.967 | 0.958 | 0.01 | 0.967 |  | 0.967 | 0.964 | 0.96 | 0.003 | 0.964 | KNN |

**Additional file 1: Table S4 |** **Accuracy of different machine learning algorithms and our deep-learning-based approach by PCA dim = 90.** accuracy on the task of disease-normal classification (right) and the task of tissue type identification (left).

| Disease classification accuracy (PCA dim=90) | | | | |  | Tissue classification accuracy (PCA dim = 90) | | | | | |
| --- | --- | --- | --- | --- | --- | --- | --- | --- | --- | --- | --- |
| **max** | **median** | **min** | **std** | **mean** |  | **max** | **median** | **min** | **std** | **mean** | **Model** |
| 0.994 | 0.992 | 0.989 | 0.002 | 0.992 |  | 0.988 | 0.986 | 0.985 | 0.002 | 0.986 | Ours |
| 0.982 | 0.981 | 0.976 | 0.003 | 0.98 |  | 0.982 | 0.979 | 0.976 | 0.002 | 0.979 | SVC |
| 0.983 | 0.979 | 0.976 | 0.003 | 0.979 |  | 0.983 | 0.979 | 0.976 | 0.003 | 0.98 | LR |
| 0.967 | 0.959 | 0.956 | 0.005 | 0.961 |  | 0.967 | 0.959 | 0.956 | 0.005 | 0.961 | LDA |
| 0.915 | 0.9 | 0.89 | 0.011 | 0.903 |  | 0.915 | 0.9 | 0.89 | 0.011 | 0.903 | NBayes |
| 0.862 | 0.854 | 0.845 | 0.007 | 0.853 |  | 0.86 | 0.855 | 0.85 | 0.004 | 0.854 | DTree |
| 0.96 | 0.958 | 0.948 | 0.005 | 0.955 |  | 0.961 | 0.952 | 0.944 | 0.007 | 0.952 | RForest |
| 0.967 | 0.964 | 0.958 | 0.004 | 0.963 |  | 0.964 | 0.964 | 0.958 | 0.003 | 0.962 | KNN |

**Additional file 1: Table S5 | Accuracy of different machine learning algorithms and our deep-learning-based approach by PCA dim = 150.** accuracy on the task of disease-normal classification (right) and the task of tissue type identification (left).

| Disease classification accuracy (PCA dim=150) | | | | |  | Tissue classification accuracy (PCA dim = 150) | | | | | |
| --- | --- | --- | --- | --- | --- | --- | --- | --- | --- | --- | --- |
| **max** | **median** | **min** | **std** | **mean** |  | **max** | **median** | **min** | **std** | **mean** | **Model** |
| 0.994 | 0.992 | 0.989 | 0.002 | 0.992 |  | 0.988 | 0.986 | 0.985 | 0.002 | 0.986 | Ours |
| 0.984 | 0.981 | 0.976 | 0.003 | 0.98 |  | 0.985 | 0.981 | 0.979 | 0.002 | 0.982 | SVC |
| 0.987 | 0.98 | 0.979 | 0.004 | 0.982 |  | 0.987 | 0.982 | 0.98 | 0.003 | 0.984 | LR |
| 0.977 | 0.971 | 0.967 | 0.004 | 0.972 |  | 0.977 | 0.971 | 0.967 | 0.004 | 0.972 | LDA |
| 0.864 | 0.861 | 0.84 | 0.01 | 0.856 |  | 0.864 | 0.861 | 0.84 | 0.01 | 0.856 | NBayes |
| 0.861 | 0.853 | 0.85 | 0.005 | 0.855 |  | 0.867 | 0.852 | 0.849 | 0.007 | 0.855 | DTree |
| 0.953 | 0.945 | 0.939 | 0.006 | 0.946 |  | 0.956 | 0.947 | 0.94 | 0.007 | 0.948 | RForest |
| 0.967 | 0.961 | 0.958 | 0.003 | 0.961 |  | 0.965 | 0.962 | 0.956 | 0.004 | 0.961 | KNN |

**Additional file 1:** **Table S6 |** The number of samples in each tissue.

| **tissue/type** | **ON** | **TT** | | **OT** | **TN** | |
| --- | --- | --- | --- | --- | --- | --- |
| **Bladder** | 28 | 28 | | 362 | 362 | |
| **Breast** | 199 | 199 | | 982 | 982 | |
| **Cervix** | 13 | 13 | | 259 | 259 | |
| **Colon** | 390 | 390 | | 372 | 372 | |
| **Esophageal** | 670 | 670 | | 183 | 183 | |
| **Kidney** | 158 | 158 | | 771 | 771 | |
| **Liver** | 163 | 163 | | 295 | 295 | |
| **Lung** | 423 | 423 | | 992 | 992 | |
| **Prostate** | 154 | 154 | | 426 | 426 | |
| **Salivary** | 97 | 97 | | 460 | 460 | |
| **Stomach** | 225 | 225 | | 380 | 380 | |
| **Thyroid** | 371 | 371 | | 441 | 441 | |
| **Uterus** | 105 | | 105 | 188 | 188 |  |

**Additional file 1:** **Table S7 | True positive rate for UP and Down genes in each tissue based on equations 1 and 2.**

| **DEG** | **UP** | | | | **DOWN** | | | **True positive** | |
| --- | --- | --- | --- | --- | --- | --- | --- | --- | --- |
| **Type** | **ON/TT** | | **OT/TN** | **intersect** | **ON/TT** | **OT/TN** | **intersect** | **% UP** | **% DN** |
| Bladder | 3434 | | 5065 | 2753 | 2069 | 1533 | 922 | 65 | 51 |
| Breast | 3064 | | 3846 | 2518 | 2455 | 1845 | 1241 | 73 | 58 |
| Cervix | 2427 | | 5166 | 1938 | 1638 | 1732 | 661 | 51 | 39 |
| Colon | 4010 | | 5198 | 3222 | 1234 | 908 | 436 | 70 | 41 |
| Esophageal | 4470 | | 4785 | 3367 | 1142 | 1451 | 635 | 73 | 49 |
| Kidney | 4063 | | 3992 | 2898 | 2295 | 1374 | 990 | 72 | 54 |
| Liver | 8548 | | 6800 | 6108 | 1718 | 1426 | 1106 | 80 | 70 |
| Lung | 3628 | | 4029 | 2700 | 2152 | 1205 | 605 | 71 | 36 |
| Prostate | 4083 | | 4812 | 3708 | 1475 | 1578 | 1042 | 83 | 68 |
| Salivary | 5079 | | 5534 | 3850 | 917 | 1153 | 369 | 73 | 36 |
| Stomach | 3639 | | 4087 | 2911 | 1040 | 1515 | 684 | 75 | 54 |
| Thyroid | 4362 | | 4476 | 3637 | 1880 | 1571 | 1076 | 82 | 62 |
| Uterus | 4400 | 5019 | | 3212 | 1730 | 1228 | 553 | 68 | 37 |

**Additional file 1:** **Table S8 | CCLE_Proteomics_2020 enrichment results.** The number of cell lines that have been correctly identified by ccle proteomics enrichment analysis for each tissue and the best cell line and its P-value.

| **P-value** | **Best Cell line** | **All cell lines** | Tissue/type | **P-value** | **Best Cell line** | **All cell lines** | Tissue/type |
| --- | --- | --- | --- | --- | --- | --- | --- |
| 0.00002909 | LU65 | 38 | lung_ON_TT | 0.00200307 | T24 | 5 | bladder_ON_TT |
| 0.01612286 | VCAP | 3 | prostate_ON_TT | 0.00042712 | HCC1395 | 7 | breast_ON_TT |
| - | - | 0 | salivary_ON_TT | - | - | 0 | cervix_ON_TT |
| 0.00046934 | HGC27 | 2 | stomach_ON_TT | 0.00005258 | SKCO1 | 13 | colon_ON_TT |
| 0.00676994 | 8305C | 2 | thyroid_ON_TT | 0.00357808 | TE4 | 4 | esophageal_ON_TT |
| 0.00122518 | HEC108 | 7 | uterus_ON_TT | 0.00000017 | A498 | 6 | kidney_ON_TT |
|  |  |  |  | 0.00000011 | JHH1 | 9 | liver_ON_TT |

**Additional file 1:** **Table S9 | Space of hyperparameter of Classifier network.**

| **Name** | **parameter** | **Space Values** |
| --- | --- | --- |
| Gaussian noise | stddev | 0,0.025,0.05 |
| Building Layer 1 | Dropout Rate | 0,0.25,0.5 |
| Building Layer 1 | Number of Units | 512,1024 |
| Building Layer 1 | Activation Function | ReLU, Identity, SoftPlus, ELU |
| Building Layer 2 | Dropout Rate | 0,0.25,0.5 |
| Building Layer 2 | Number of Units | 128,256 |
| Building Layer 2 | Activation Function | ReLU, Identity, SoftPlus, ELU |
| Building Layer 3 | Dropout Rate | 0,0.25,0.5 |
| Building Layer 3 | Number of Units | 8,12,16,20,28,24,32 |
| Building Layer 3 | Activation Function | ReLU, Identity, SoftPlus, ELU |
| Bottleneck Layer | Dropout Rate | 0 |
| Bottleneck Layer | Number of Units | 8,12,16,20,28,24,32 |
| Bottleneck Layer | Activation Function | Softmax |
| Building Layer 5 | Dropout Rate | 0,0.25,0.5 |
| Building Layer 5 | Number of Units | 256,512 |
| Building Layer 5 | Activation Function | ReLU, Identity, SoftPlus, ELU |
| - | Batch Size | 32,64,128 |
| - | Number of epoch | 150,200,250 |

**Additional file 1: Table S10 | Space of hyperparameter of Transferor network.**

| **Name** | **parameter** | **Space Values** |
| --- | --- | --- |
| Building Layer 1 | Number of Units | 256,512,1024 |
| Building Layer 1 | Activation Function | ReLU, Identity, SoftPlus |
| Building Layer 1 | Dropout Rate | 0,0.25,0.5 |
| Building Layer 2 | Number of Units | 128,256 |
| Building Layer 2 | Activation Function | ReLU, Identity, SoftPlus |
| Building Layer 2 | Dropout Rate | 0,0.25,0.5 |
| Bottleneck Layer | Number of Units | 8,12,16,20,28,24,32 |
| Bottleneck Layer | Activation Function | ReLU, Identity, SoftPlus |
| Bottleneck Layer | Dropout Rate | 0,0.25,0.5 |
| Building Layer 4 | Number of Units | 128,256 |
| Building Layer 4 | Activation Function | ReLU, Identity, SoftPlus |
| Building Layer 4 | Dropout Rate | 0,0.25,0.5 |
| Building Layer 5 | Number of Units | 1024,256,512 |
| Building Layer 5 | Activation Function | ReLU, Identity, SoftPlus |
| Building Layer 5 | Dropout Rate | 0,0.25,0.5 |
| - | Batch Size | 32,64,128,256 |
| - | Number of epoch | 100,150,200,250 |
