# Supplementary material for: DeeP4med: deep learning for P4 medicine to predict normal and cancer transcriptome in multiple human tissues
Source: BMC Bioinformatics. 2023 Jul 4;24:275. doi: 10.1186/s12859-023-05400-2 (PMC10320882; doi:10.1186/s12859-023-05400-2)
Supplement: Supplementary file 2 — Additional file 2. Dataset (gene expression matrix of different tissues), DEGs and enrichment analysis results, common and important genes between different matrices. [file 12859_2023_5400_MOESM2_ESM.docx]

Note: The datasets analysed during the current study and its supplementary information files are available in the google drive repository,

<https://drive.google.com/drive/folders/1lMMQdMXsHT8fcP9Mz9sb6NpyFByI7rcj?usp=share_link>

The code is available from the corresponding author upon reasonable request ([stahmasebian@gmail.com](mailto:stahmasebian@gmail.com)).

[**https://drive.google.com/drive/folders/1lMMQdMXsHT8fcP9Mz9sb6NpyFByI7rcj?usp=share_link**](https://drive.google.com/drive/folders/1lMMQdMXsHT8fcP9Mz9sb6NpyFByI7rcj?usp=share_link)

**DeeP4med: deep learning for P4 medicine to predict normal and cancer transcriptome in multiple human tissues**

**Roohallah Mahdi Esferizi^1,+^, Behnaz Haji Molla Hoseyni^2,+^, Amir Mehrpanah^3^, Yazdan Golzade^4^, Ali Najafi^5^, Fatemeh Elahian^1^, Amin Zadeh Shirazi^6^, Guillermo A. Gomez^6^, and Shahram Tahmasebian^7,^***

^1^ Department of Medical Biotechnology, School of Advanced Technologies, Shahrekord University of
Medical Sciences, Shahrekord, Iran ([roohallah1435@gmail.com](mailto:roohallah1435@gmail.com), [dr.elahian@yahoo.com](mailto:dr.elahian@yahoo.com))
^2^ Laboratory of Systems Biology and Bioinformatics (LBB), University of Tehran, Tehran, Iran
([hoseyni.sb@gmail.com](mailto:hoseyni.sb@gmail.com))
^3^ Faculty of Mathematics, Shahid Beheshti University, Tehran, Iran ([a.mehrpanah@mail.sbu.ac.ir](mailto:a.mehrpanah@mail.sbu.ac.ir))
^4^ Department of Mathematics, Faculty of Basic Sciences, Iran University of Science and
Technology,(IUST). Tehran, Iran. ([Yazdan.golzade@gmail.com](mailto:Yazdan.golzade@gmail.com))
^5^ Molecular Biology Research Center, Systems Biology and Poisonings Institute, Baqiyatallah University of Medical Sciences, Tehran, Iran. ([najafi74@bmsu.ac.ir](mailto:najafi74@bmsu.ac.ir))
^6^ Centre for Cancer Biology, SA Pathology and University of South Australia, Adelaide, SA 5000,
Australia ([amin.zadeh_shirazi@mymail.unisa.edu.au](mailto:amin.zadeh_shirazi@mymail.unisa.edu.au), [Guillermo.Gomez@unisa.edu.au](mailto:Guillermo.Gomez@unisa.edu.au))
^7^ Cellular and Molecular Research Center, Basic Health Sciences Institute, Shahrekord University of
Medical Sciences, Shahrekord, Iran ([stahmasebian@gmail.com](mailto:stahmasebian@gmail.com))
+ Authors Contributed Equally
 * Corresponding author

**This includes:**

**SUPPLEMENTARY INFORMATION PART 1:**

The expression matrix for each of the tissues in OT_TN and ON_TT states is produced by the model. In each matrix, the names of genes are in rows and healthy and cancerous samples are in columns. In each expression matrix, 18151 genes have been identified.

**SUPPLEMENTARY INFORMATION PART 2:**After carrying out DEGs analysis by iDEP, the results for all tissues are in different sheets of Excel files. Its columns contain the Ensembl ID of each gene, Symbol, logFC and adj-P-Val respectively. Also, the results of PCA analysis for all tissues are in the PDF file.

**SUPPLEMENTARY INFORMATION PART 3:**

Venn diagrams to show common genes separately in up and down states in each tissue between OT_TN & ON_TT.

**SUPPLEMENTARY INFORMATION PART 4:**

Enrichment results separately for each tissue in OT_TN & ON_TT. four types of enrichment analyses were performed for each tissue, which are: (1) Gene ontology (GO)_biological process. (2) Cancer Cell Line Encyclopedia (CCLE) Proteomics. (3) Kyoto Encyclopedia of Genes and Genomes (KEGG) pathway. (4) ChIP Enrichment Analysis (ChEA). These results are shown for each tissue separately, in different Excel sheets. Also, the bar plot of these enrichments is shown in the PDF file.

**SUPPLEMENTARY INFORMATION PART 5:**

This folder contains the Venn diagrams and the list of common DEGs genes obtained by comparing the three types of matrices with each other (OT_TN & ON_TT & original)

**SUPPLEMENTARY INFORMATION PART 6:**

This folder contains the Venn diagrams, and the lists of genes are the most important because they exist in all three matrices.
